# HBV and HIV/HBV Infected Patients Have Distinct Immune Exhaustion and Apoptotic Serum Biomarker Profiles

**DOI:** 10.20411/pai.v4i1.267

**Published:** 2019-02-13

**Authors:** Mohamed Tarek M. Shata, Enass A. Abdel-hameed, Susan D. Rouster, Li Yu, Meina Liang, Esther Song, Mark T. Esser, Norah Shire, Kenneth E. Sherman

**Affiliations:** 1 Internal medicine; University of Cincinnati; Cincinnati, Ohio; 2 MedImmune; Gaithersburg, Maryland; 3 MedImmune; 121 Oyster Point Boulevard; South San Francisco, California; 4 AstraZeneca; Gaithersburg, Maryland

**Keywords:** HBV, HIV, coinfection HBV/HIV, Immune exhaustion markers, sFas, PD-1, TNF-α

## Abstract

**Background:**

Hepatitis B virus (HBV) infection is a leading cause of chronic hepatitis, liver cirrhosis, and hepatocellular carcinoma worldwide. Due to their shared routes of transmission, approximately 10% of HIV-infected patients worldwide are chronically coinfected with HBV. Additionally, liver disease has become a major cause of morbidity and mortality in HBV/HIV coinfected patients due to prolonged survival with the success of antiretroviral therapy. The relationship between immune exhaustion markers (PD-1/PD-L1) and apoptotic markers such as Fas/FasL, TGFβ1, TNF-α, and Th1/Th2 cytokines are not clearly delineated in HBV/HIV coinfection.

**Methods:**

Levels of soluble Fas/FasL, TGFβ1, TNF-α, and sPD-1/sPD-L1 as well as Th1 and Th2 cytokines were evaluated in the sera of HBV-monoinfected (n = 30) and HBV/HIV-coinfected (n = 15) patients and compared to levels in healthy controls (n = 20).

**Results:**

HBV-monoinfected patients had significantly lower levels of the anti-inflammatory cytokine IL-4 (*P* < 0.05) and higher levels of apoptotic markers sFas, sFasL, and TGFβ-1 (*P* < 0.001) compared to healthy controls. Coinfection with HIV was associated with higher levels of sFas, TNF-α, and sPD-L1 (*P* < 0.005), and higher levels of the pro-inflammatory cytokines IL-6, IL-8, and IL-12p70 (*P* < 0.05) compared to healthy controls. Patients with HBV infection had a unique biomarker clustering profile comprised of IFN-γ, IL12p70, IL-10, IL-6, and TNF-α that was distinct from the profile of the healthy controls, and the unique HIV/HBV profile comprised GM-CSF, IL-4, IL-2, IFN-γ, IL12p70, IL-7, IL-10, and IL-1β. In HBV monoinfection a significant correlation between sFasL and PD1(r = 0.46, *P* = < 0.05) and between sFas and PDL1 (r = 0.48, *P* = <0.01) was observed.

**Conclusion:**

HBV-infected and HBV/HIV-coinfected patients have unique apoptosis and inflammatory biomarker profiles that distinguish them from each other and healthy controls. The utilization of those unique biomarker profiles for monitoring disease progression or identifying individuals who may benefit from novel immunotherapies such as anti-PD-L1 or anti-PD-1 checkpoint inhibitors appears promising and warrants further investigation.

## INTRODUCTION

Hepatitis B virus (HBV) infection is a leading cause of chronic hepatitis, liver cirrhosis, and hepatocellular carcinoma (HCC) worldwide. It is estimated that 257 million people are infected with chronic HBV with approximately 3300 new cases per year in the United States alone. HBV is a non-cytopathic virus; therefore, liver damage — leading to fibrosis, cirrhosis, and HCC — is due to the immune response to viral infection [1, 2].

Due to their shared routes of transmission, HIV is common among patients infected with HBV. Approximately, 10% of HIV-infected patients worldwide are thought to be coinfected with HBV. Additionally, liver disease has become a major cause of morbidity and mortality in HBV/HIV coinfected patients due to prolonged survival with success in antiretroviral therapy (ART) [3, 4].

The development of chronic HBV infection is influenced by several factors: (1) Exhausted or tolerant HBV-specific CD8+ T cells [5, 6]; (2) Suppressed CD4+ T-cell responsiveness mediated by dendritic cell impairment[7-9] or upregulation of PD-1 expression [10, 11]; (3) Impaired innate immune responses through decreased natural killer cell function [12]; (4) Increased number of regulatory T cells (T_reg_) leading to suppression of HBV-specific T-cell responses and decreased viral clearance [13, 14]; and (5) Induced apoptosis of Fas-expressing lymphocytes through activation of Fas/FasL pathways in Kupffer cells prompting immune tolerance to HBV and overcoming HBV clearance by the induced Fas/FasL apoptosis of hepatocytes [15].

Immune dysregulation is also thought to be a major factor leading to chronic HBV infection and the resulting liver diseases and carcinogenesis. A strong Th1-like immune response, characterized by high levels of functional cytolytic T lymphocytes (CTL), correlates with virus control and functional cures. A Th2-like inflammatory response is associated with viral persistence and progression of immunopathogenesis of HBV [16, 17]. The interactions among the different immunological pathways are complicated. For example, the pro-inflammatory cytokine IL-8 has been shown to be a marker of liver damage and can also inhibit the antiviral activity of IFN-α [18-20]. TGF-β1 is a major regulator of liver fibrosis; it can downregulate Th1 responses [21-26] and has been implicated in the development of HCC [27, 28]. Determining serum levels of cytokines and other immunoregulatory proteins may provide a measure to determine the likelihood of resolving an HBV infection or the risk of developing HCC.

In addition to the Th1/Th2 cytokine regulations, the PD-1/PD-L1 mechanism plays an important role in T-cell homeostasis, and it is involved in the regulation of anti-viral and anti-tumor immune response. PD-1 is expressed on both T and B cells in response to most immune challenges. The PD-1 protein is a negative regulator of T-cell activation [29]. The PD-1 signalling pathway is activated by binding PD-1 to its cognate receptors PD-L1 and/or PD-L2 [29, 30], which induces the inhibitory signal to impede the proliferation of T and B cells and to maintain peripheral tolerance [29, 30]. On chronically stimulated Ag-specific T cells, PD-1 expression remains high, leading to an impaired response to stimuli. On hepatic T cells, PD-1 is expressed on the cell surface and is an important checkpoint molecule which can transduce co-inhibitory signals to immuno-competent cells and exert immunosuppressive roles. In addition to the membrane-bound PD-1 on T cells, there is a soluble PD-1 (sPD-1) [31] which is encoded by an alternative splice variant PD-1 Delta ex3 that lacks the transmembrane domain of the PD-1 molecule and can enhance anti-tumor immune responses [32-34]. In inflamed liver cytoplasmic expression of PD-L1 has been detected and may represent intracellular stores of PD-L1, which could be expressed upon proper stimulation on the cell surface or released in a soluble form into the microenvironment to suppress the anti-HBV immune responses [34-37]. PD-1 expression on CD8+ T cells correlates with viral load in patients with chronic HBV [38]. *In vitro* blocking of PD-1/PD-L1 interactions results in functional restoration of HBV-specific CD8+ T cells [39]. During HBV infection, higher levels of sPD-1 have been associated with immune tolerance and increased prevalence of HCC [40, 41]. These data suggest that monitoring sPD-1 or PD-L1 levels during infection may have prognostic value, and that PD-1 or PD-L1 may be an attractive target for restoring anti-HBV-specific T-cell responses in patients to either control or eradicate HBV.

The Fas/FasL system also plays an important role in the regulation of the immune response to HBV in the liver and the apoptosis of infected hepatocytes. HBV-specific CD8^+^ T cells can kill HBV-infected hepatocytes via the perforin/granzyme mechanism of killing or by the Fas/FasL mediated mechanism of killing. However, death of HBV-infected hepatocytes is thought to occur primarily through Fas-mediated killing. Soluble Fas (sFas) and soluble Fas ligand (sFasL) have been shown to inhibit hepatocyte apoptosis [42-44] allowing for the persistence of HBV in hepatocytes [45]. The Fas pathway is also involved in the apoptosis of activated T cells as a mechanism to maintain peripheral tolerance. A high level of Fas expression in HBV infected hepatocytes is thought to delete HBV-specific T cells leading to chronic infection and the development of HCC [46]. Interestingly, human HCC cell lines have been shown to be resistant to Fas-mediated apoptosis [47]. Soluble Fas and sFasL have much higher in cirrhosis and patients with HCC compared to normal controls [46].

In HBV/HIV-coinfected patients, there is acceleration of the immunologic and clinical progression of HIV infection with an increased risk of hepatotoxicity. Additionally, HIV infection increases the risk of hepatitis events, cirrhosis, and end-stage liver disease related to chronic HBV infection[48]. The immunological profiles associated with high morbidity in HBV/HIV coinfected patients are not fully understood. In this cross-sectional study we measured the serum levels of immunologic (Th1/Th2 and pro-inflammatory) cytokines and immunoregulatory proteins (sFasL, sFas, sPD-L1, and sPD-1) to test the hypothesis that their levels differ among individuals with chronic HBV or HIV/HBV coinfections and healthy controls.

## MATERIAL AND METHODS

### Enrolled Patients

Thirty HBV-monoinfected patients and 15 HBV/HIV-coinfected patients from the University of Cincinnati Infectious Disease Center (UC IDC) and Hepatology clinics were previously evaluated in a retrospective study to determine HBV status [49]. To diagnose HBV, serological diagnoses of HBV infection (HBsAg) were detected by ELISA (BioChain, Hayward, CA). In some cases, HBV DNA was quantified using real-time PCR performed in triplicate and compared to a standard panel to determine viral titer (lower limit of detection [50] of 100 IU/mL). To diagnose HIV, serological diagnoses of HIV were performed. When available, HIV RNA levels were determined by either qualitative or quantitative reverse transcriptase polymerase chain reactions (RT-PCR) obtained from clinical databases. Healthy controls were selected from volunteer lab workers with no history of HIV or HBV and negative serological markers for both HIV and HBV. Stored sera from healthy controls (20) were used as controls.

### Multiplex Assay

The Human MILLIPLEX assay (EMD Millipore Corporation, Billerica, MA) was employed to measure serum concentrations of 13 immune markers: GM-CSF, IFNγ, IL-1β, IL-2, IL-4, IL-5, IL-6, IL-7, IL-8, IL-10, IL-12 (p70), IL-13, TNF-α, a 2-plex assay to measure apoptosis markers sFas and sFasL and a single-plex assay to measure TGF-β1. Mean fluorescence intensity (MFI) analyte-specific signals were interpolated using standard curves and analyte concentrations were calculated using a 5-parameter logistic curve fitting algorithm on the Bio-Plex Manager 6.1.

Serum sPD-1 was measured using a sandwich ELISA assay (R&D, Minneapolis, MN). Mean absorbance sPD-1 values were measured on a Biotek reader (Biotek, Winooski, VT) and sPD-1 concentrations (pg/mL) were calculated from a standard curve generated using a 4-parameter logistic curve fitting model. The dynamic range of the assay was 156 to 10,000 pg/mL.

### Soluble PD-L1 Assay

An ECL immunoassay was used to measure the concentration of sPD-L1 in human serum using the MSD platform that has been previously described [51]. In brief, standards, controls, and test samples were incubated with biotinylated anti-PD-L1 capture antibody clone 2.7 A4 (MedImmune) that was immobilized on an MSD streptavidin-coated 96-well plate. After incubation, unbound material was washed away, and captured sPD-L1 was detected by addition of anti-PD-LI primary detection antibody clone 130021 (R&D Systems). Unbound primary detection antibody was removed by washing. Bound primary detection antibody was detected by the addition of ruthenium-labeled secondary detection antibody (MSD), and the signal for each plate well was measured by an MSD Sector. The assay quantitation range was 15.6 to 1000 pg/mL.

Concentrations below the lower limit of quantitation for all assays were assigned a value of one-half the lower limit of the quantitation value for statistical comparisons. Samples with values greater than the upper limit of quantitation were further diluted, and dilution-corrected analyte concentrations were calculated accordingly.

### Statistical Analyses

A general linear regression model was used to compare cytokine levels of individuals with disease to healthy controls adjusted for age, sex, and race effects. For each cytokine, to assess the difference between disease groups, the age, sex, and race were included in the model as covariates to evaluate potential effects on cytokine levels due to these covariates. If any of the covariates displayed statistical significance (*P* value ≤ 0.05), the covariate would be included in the final general linear regression model, in addition to disease groups, to adjust for the effect due to covariates. The Spearman correlation coefficient was used to assess correlation between paired cytokine levels. The cluster analysis was applied to the correlation coefficient matrix to identify clusters of cytokines with average correlation coefficient values ≥ 0.55. Statistical software packages including SAS 9.4 and R 3.4.3 were used for analysis.

## RESULTS

### Characterization of Enrolled Individuals

Demographics for the 30 patients with chronic HBV infection, the 15 with HBV/HIV coinfection and the 20 healthy controls are listed in Table 1. The average age for chronic HBV patients was 41.1 years, 45.1 years for HBV/HIV-coinfected patients, and 26.9 years for healthy controls. Patients who were HBV monoinfected and HBV/HIV coinfected were significantly (*P* < 0.001) older than the healthy controls. HBV-infected patients were predominantly Asian (40%), while HBV/HIV-coinfected patients and healthy controls were predominantly white (73%, and 55%, respectively). Median alanine aminotransferase (ALT) levels were 40 U/L in chronic HBV patients and 115.5 U/L in HBV/HIV-coinfected patients. Median aspartate transaminase (AST) levels were 29.5 U/L in chronic HBV patients and 109.5 U/L in HBV/HIV-coinfected patients.

**Table 1.** Participant demographics

Participants	HBV Monoinfection N = 30	HBV/HIV Coinfection N = 15	Healthy Controls N = 20	*P* value HBV&HBV/HIV	*P* value HBV&HC	*P* value HC& HBV/HIV
Age (Avg ± STD) Median (IQR)	41.1 ± 14.95 35 (27.5)	45.1 ± 7.9 45 (40)	26.9 ± 6.1 26 (22)	NS	< 0.001	< 0.001
Race						
Black	8	3	5			
White	9	11	11			
Asian	12	0	4			
Hispanic	0	1	0			
ALT U/L (Avg ± STD) Median (IQR)	47.75 ± 47.8 40 (23.25)	153.7 ± 132.8 115.5 (59.25)	ND	< 0.01	< 0.01	< 0.01
AST U/L (Avg ± STD) Median (IQR)	31.1 ± 26.8 29.5 (21)	108.1 ± 73.4 109.5 (41)	ND	< 0.01	< 0.01	< 0.01
HBV log copies/mL (Avg ± STD) Median (IQR)	4.98 ± 2.3 4.65 (3.46)	6.4 ± 1.2 6.47 (5.3)	ND	NS	ND	ND
*% of patient with Cirrhosis	25%	25%	0	NS	ND	ND
HIV log copies/mL (Avg ± STD) Median (IQR)	NA	6501 ± 9369 4440 (2.7)	ND	ND	ND	ND
% of patients with detectable HIV	NA	91%	ND	ND	ND	ND
CD4 count (Avg ± STD) Median, IQR	ND	536 ± 285 520 (236)	ND	ND	ND	ND

ND = not done, NS = Not significant

* AST/ALT ratio (AAR) was used as marker of cirrhosis (> 1) for the enrolled subjects

ALT and AST were significantly higher (*P* < 0.01) in HBV/HIV-coinfected patients compared to those with HBV monoinfection, and compared to the normal levels for ALT (10-40 U/L) and AST (10-34 U/L). At the time of serum sample collection, HIV RNA was detectable by either a qualitative or a quantitative assay in 10 of 11 (91%) HBV/HIV-coinfected patients tested, and below the levels of detection in 1 (9%) of the HBV/HIV patients tested. Among the HBV/HIV individuals with quantitative HIV viral loads, the median HIV RNA level was 4.4 × 10^3^ copies/mL. CD4 counts were available for 8 of 15 coinfected patients, with an average of 536 ± 285.85. No significant differences were observed between HIV-controlled and HIV-uncontrolled patients or the types of treatment. We utilized the AST/ALT ratio (AAR) as a marker of cirrhosis (> 1) for the enrolled patients [50, 52-55]. For those with HBV alone, 4 of 16 had indications of cirrhosis, and 3 of 12 of the coinfected group had indications of cirrhosis. At the time of sample collection, only 15% of those with HBV monoinfection were receiving anti-HBV therapy, and 60% of those with HBV/HIV coinfection were receiving antiretroviral therapy containing anti-HBV-active agents. The average HBV DNA level was not significantly different among persons with HBV monoinfection, 4.98 × 10^3^ ± 2.3 × 10^3^ IU/mL, compared to 6.4 × 10^3^ ± 1.2 × 10^3^ IU/mL among persons with HBV/HIV coinfection.

### Distinct Serum Biomarker Profiles in HBV Versus HBV/HIV Infected Individuals

**Figure 1.**
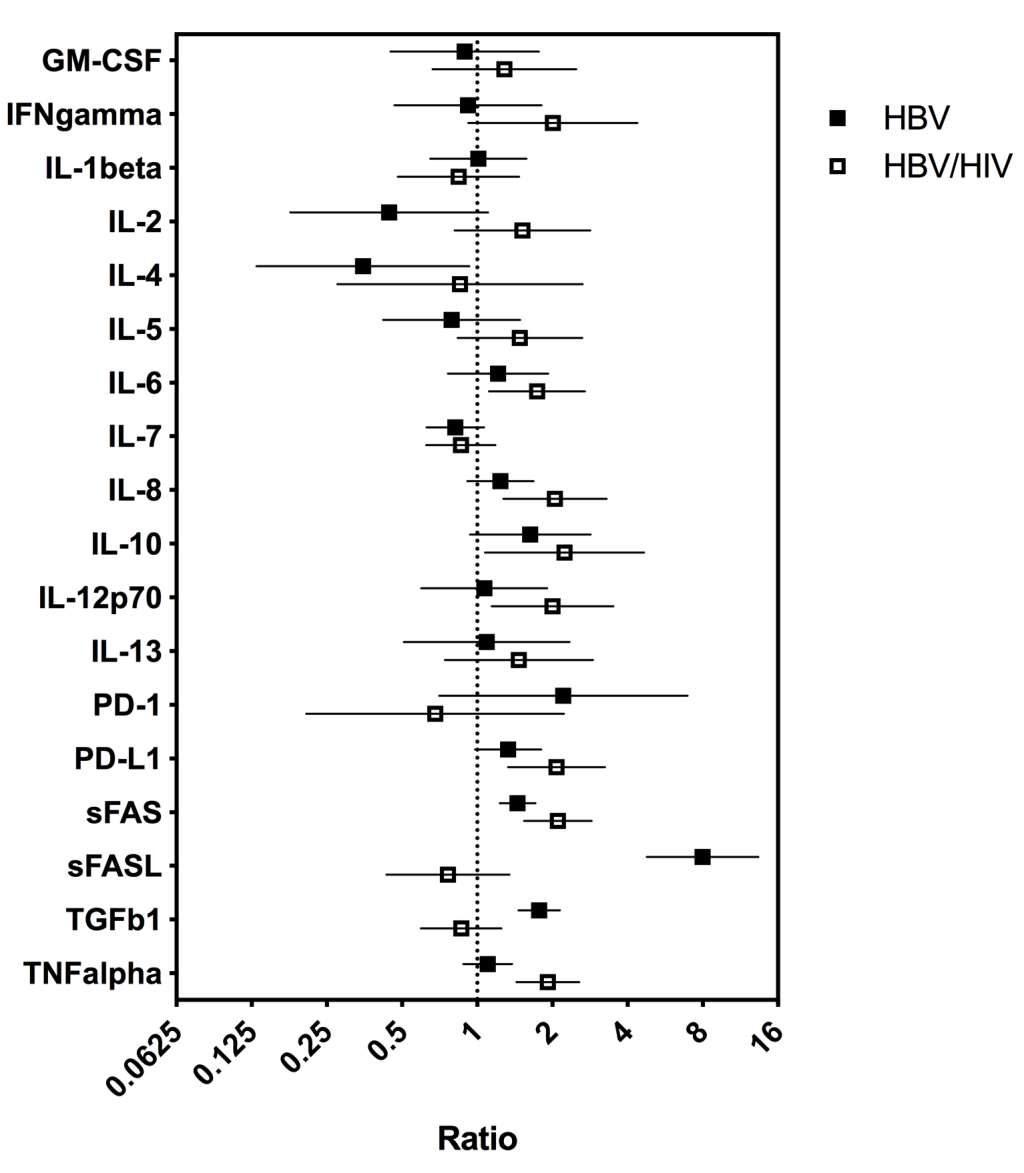
**Fold-differences in biomarker values between HBV- and HBV/HIV-infected patients relative to healthy controls.** Difference from healthy control is statistically significant if 95% CI does not cross “1”. Analysis adjusted for covariates including age, gender, and race when effects of covariates were statistically significant (*P* < 0.05).

**Figure 2.**
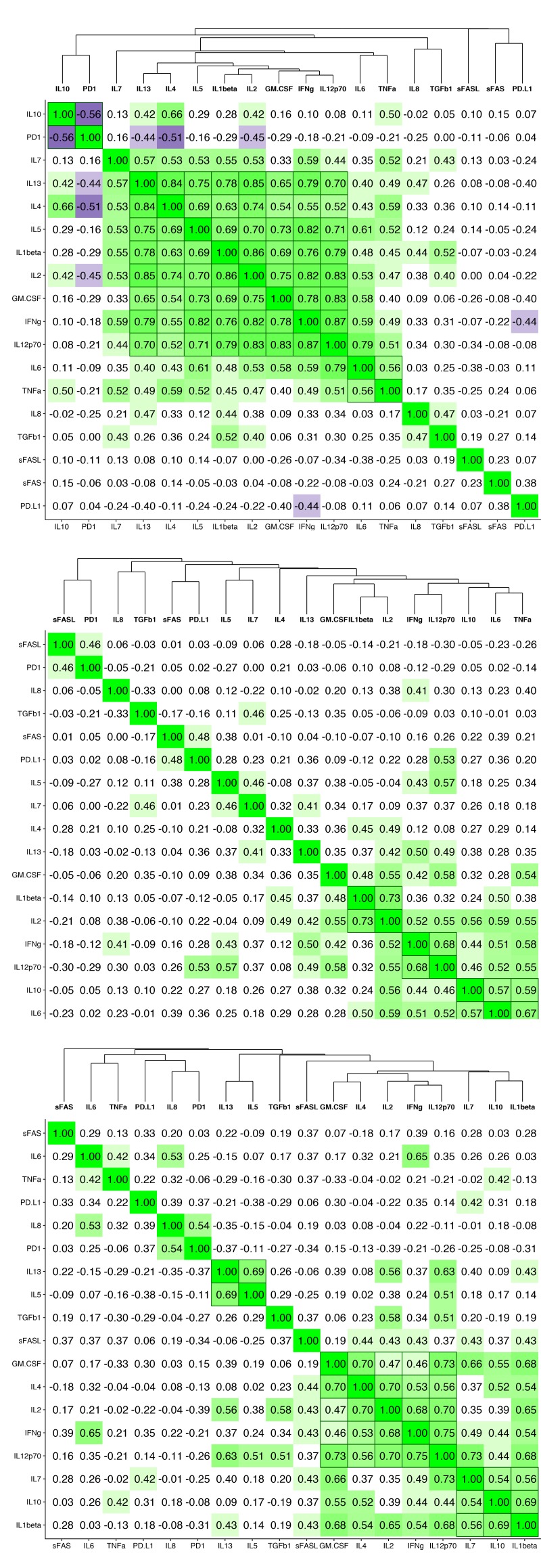
**Cluster correlation biomarker profiles**. Correlation coefficients of the immune and apoptotic biomarkers are shown for (A) healthy controls, (B) HBV-monoinfected patients and (C) HBV/HIV-coinfected patients.

**Supplementary Figure 1.**
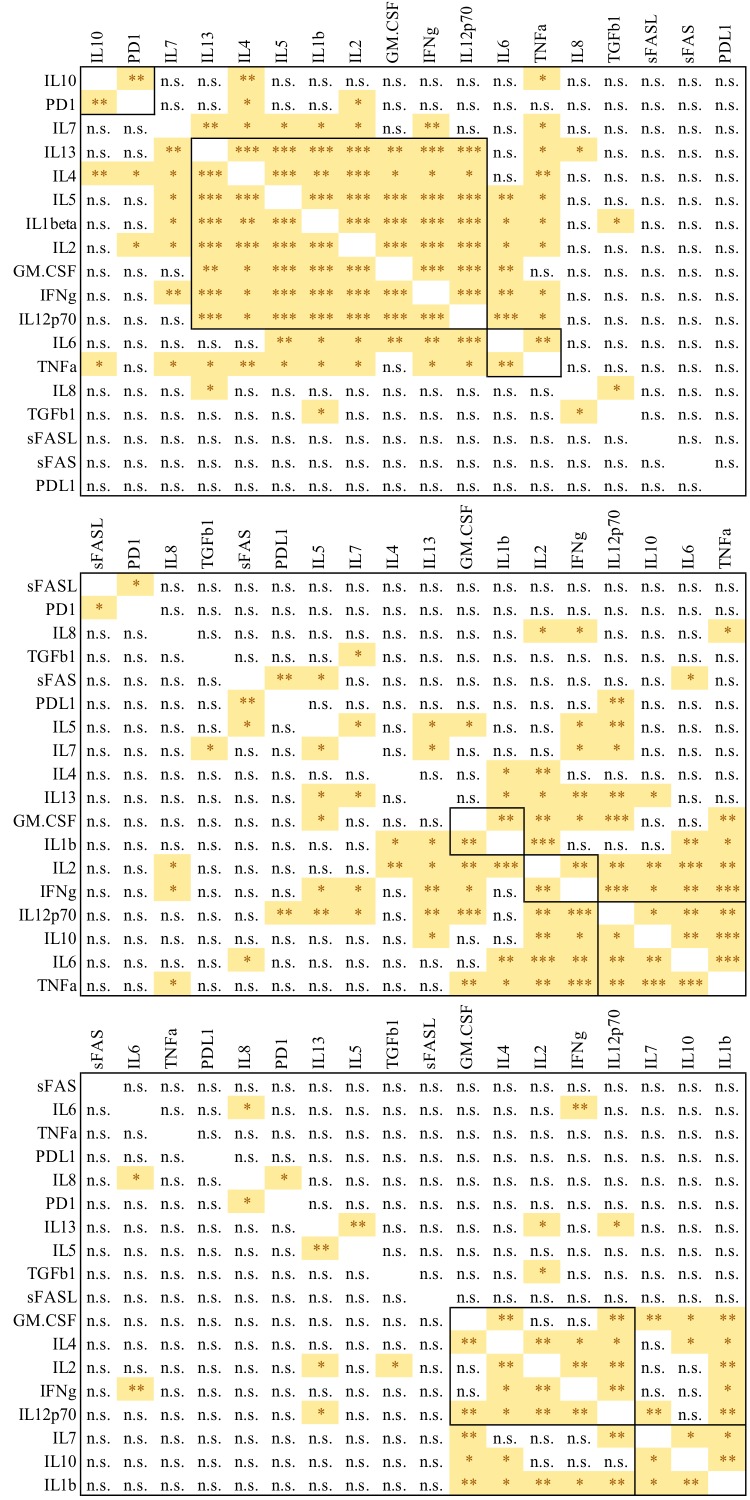
*P* Values for Figure 2. Correlation coefficients P values for biomarker levels are shown for (A) healthy controls, (B) HBV-monoinfected patients and (C) HBV/HIV-coinfected patients, *P* value<0.05 (*), <0.01(**), <0.001(***), >0.05(n.s.)

To determine whether serum cytokine and inflammatory biomarker levels were different between HBV- and HBV/HIV-infected patients compared to one another and to healthy controls we tested the sera in a multiplex cytokine panel assay, an sPD-1 ELISA, and a proprietary ECL-based sPD-L1 assay (Table 2). Since there were differences in age, gender, and race between the 3 groups, a statistical model was developed to determine if the biomarker levels were different between the groups when adjusting for the covariates. The fold-differences between patients with HBV and controls and patients with HBV/HIV and controls and the 95% confidence intervals are shown in Figure 1. Comparing the fold-differences of the geometric means of the various bio-markers in the individuals with HBV or HBV/HIV to the healthy controls revealed statistically lower levels of IL-4 (*P* = 0.036) and significantly higher levels of apoptotic markers such as sFas, sFasL, and TGFβ-1 (*P* < 0.001) for HBV-infected patients compared to healthy controls (Figure 1). Coinfection with HIV was associated with higher levels of sFas, TNF-α, and sPD-L1 compared to healthy controls (*P* < 0.005). Additionally, multiple inflammatory cytokines including IL-6, IL-8, IL-10, IL-12p70 were significantly higher (*P* < 0.05) compared to controls (Figure 1). Interestingly, sFasL levels were much higher in HBV-monoinfected patients compared to healthy controls and HBV/HIV-infected patients (Figure 1). To adjust for covariates between groups including age, gender, and race, regression analyses for biomarkers with statistically significant differences less than *P*=0.05 were examined. Regression analysis determined that the only significant covariate was age for sFas levels but not for gender and race. To evaluate the relationship and the potential interactions among the biomarkers we examined the cluster correlation coefficient in the 3 groups. The cluster coefficient analyses revealed a distinct profile for HBV monoinfection with GM-CSF-IL4-IL2-IFNγ-IL12p70; IL-10- IL-6-TNF-α) grouping together and a different, unique profile for HBV/HIV infection with (GM-CSF- IL-4- IL-2- IFN-γ- IL12p70; IL-7- IL-10 -IL1-β grouping together. The profiles of both HBV and HBV/HIV patients are distinctly different from the profile of the healthy controls with IL10-PD-1; IL13-IL4-IL5- IL1b- IL2-GM-CSF- IFN-γ-IL12p70; IL-6- TNF-α grouping together (Figure 2). In the group with HBV monoinfection a significant correlation between sFasL and PD1(r = 0.46, *P* = <0.05) and between sFas and PDL1 (r = 0.48, *P* = <0.01) was observed (Figure 1 and Supplementary Figure 1).

**Table 2.** Geometric mean, standard deviation, and *P* value for comparison of HBV mono-infected and HBV/HIV co-infected subjects to healthy controls

Cytokine	Healthy Controls (N = 20)	HBV Monoinfection (N = 30)			HBV/HIV Coinfection (N = 15)		
	Geometric Mean (SD)	Geometric Mean (SD)	*P*-Value	q-Value ^a^	Geometric Mean (SD)	*P*-Value	q-Value ^a^
GM-CSF	142.6 (224.8)	94.6 (69.5)	0.7371	0.8420	120.3 (127.5)	0.4488	0.5814
IFN-γ	19.0 (26.2)	17.0 (18.9)	0.8026	0.8420	31.6 (32.6)	0.0783	0.2004
IL-1β	3.8 (3.6)	3.9 (4.1)	0.9597	0.9597	3.2 (3.0)	0.5358	0.6430
IL-2	6.2 (5.8)	2.4 (8.4)	0.0801	0.2004	6.8 (5.7)	0.1863	0.3353
IL-4	12.0 (29.8)	4.2 (22.7)	0.0360	0.1178	10.2 (18.5)	0.7762	0.8420
IL-5	3.3 (4.0)	2.6 (2.3)	0.4522	0.5814	5.0 (2.4)	0.1702	0.3225
IL-6	3.1 (2.0)	3.8 (2.7)	0.4043	0.5800	5.4 (3.0)	0.0161	0.0680
IL-7	20.2 (14.6)	15.2 (4.9)	0.1315	0.2959	15.8 (7.6)	0.3448	0.5397
IL-8	12.6 (6.5)	15.6 (17.2)	0.1627	0.3225	25.7 (29.9)	0.0052	0.0267
IL-10	9.8 (9.7)	13.2 (14.7)	0.0835	0.2004	16.5 (21.8)	0.032	0.1152
IL-12p70	6.0 (6.0)	6.4 (4.8)	0.8186	0.8420	12.0 (5.7)	0.017	0.0680
IL-13	7.7 (7.4)	6.7 (11.8)	0.8163	0.8420	7.7 (5.9)	0.2624	0.4498
TNF-α	14.9 (5.2)	16.4 (10.8)	0.3878	0.5800	28.5 (16.8)	<.0001	< .0001
sFas	7,114.2 (2682.4)	10,129.1 (3511.4)	< .0001	< .0001	18,384.1 (9775.4)	< .0001	< .0001
sFasL	9.2 (76.7)	73.2 (29.1)	< .0001	< .0001	7.0 (10.9)	0.3412	0.5397
TGF-β1	22,135.1 (7,812.3)	34,987.2 (7331.2)	< .0001	< .0001	16,992.7 (7756.2)	0.4189	0.5800
sPD-1	389.9 (2569.2)	862.7 (3972.1)	0.169	0.3225	264.5 (2308.1)	0.5087	0.6315
sPD-L1	176.3 (104.7)	234.5 (232.8)	0.0652	0.1956	366.4 (367.1)	0.0025	0.0150

^a^ q-value is for false discovery rate adjusted *P* value

### Correlation Among Clinical Parameters and Immunological Markers

There are no significant correlations among HBV levels, HIV levels, CD4 counts, presence of cirrhosis, and the different immunological markers tested. However, there are significant positive correlations between ALT levels and IL-4 (*P* = 0.04, r = 0.39) and TNFα (*P* = 0.009, r = 0.48), and significant negative correlations with TGF-β (*P* = 0.007, r = -0.49). Additionally, there are significant positive correlations between AST and IL-10 (*P* = 0.03, r = 0.41), IL-4 (*P* = 0.01, r = 0.47), IL-8 (*P* = 0.01, r = 0.48), and TNFα (*P* = 0.008, r = 0.59), and significant negative correlations with TGF-β (*P* = 0.0001, r = -0.66).

## DISCUSSION

In this cross-sectional study, we examined the serum immunological profiles and apoptotic markers of patients with HBV monoinfection (n = 30) or HBV/HIV coinfection (n = 15) and healthy controls (n = 20). Significantly higher levels (*P* < 0.001) of sFas (CD95; tumor necrosis factor receptor superfamily member 6, TNFR-SF6) were observed in the HBV and HBV/HIV-coinfected patients compared to the controls. Fas receptor is a member of the death receptor family, a sub-family of the tumor necrosis factor receptor superfamily. Fas is activated through oligomerization upon binding of FasL or the agonistic anti-Fas antibody. This causes formation of the death-inducing signaling complex (DISC), and the downstream activation of the death signal pathway, a cascade of interleukin-1β-converting enzyme-like cysteine proteases [56]. Fas-induced apoptosis is involved in the cytotoxic activity of T cells and natural killer cells [57]. The Fas/FasL pathway plays an important role in controlling the local inflammatory response during viral infection [58]. Increasing evidence suggests that the Fas receptor is a mediator of apoptosis-independent processes such as induction of activating and pro-inflammatory signals [57-61]. Previous studies reported enhanced Fas system-mediated hepatocyte apoptosis in HIV- and HBV-associated chronic inflammation. In HBV viral hepatitis, liver-infiltrating lymphocytes that recognize the viral antigen on hepatocytes become activated and express FasL. In contrast, hepatocytes exhibit enhanced Fas expression and become susceptible to FasL-mediated death. Thus, Fas-mediated apoptosis plays an important role in HBV viral hepatitis and HIV infection [24, 62, 63]. It is interesting to note that the sFasL levels in HBV/HIV-coinfected patients were decreased in contrast to HBV infection (Figure 1 and Table 2). A previous study found a negative correlation between sFasL and CD4^+^ count in HIV-infected patients [64]. Since FasL is mainly expressed on CD4^+^ T cells, and in those patients with HBV/HIV coinfection the numbers of CD4^+^ T cells are known to be lower than in those with HBV monoinfection or in healthy controls [65], it is not surprising to see lower sFasL shed from exhausted and numerically decreased CD4^+^ T-cell populations. Moreover, the balance between expression of sFas and sFasL is complicated. For example, in juvenile Systemic lupus erythematosus, a distinct profile from adult SLE, there is increased sFas and reduced sFasL, notably in patients with active disease and with nephritis [66]. Additionally, Pinti *et al*, reported an increase in the production of Fas with age [67], while the production of sFasL is consistently reduced [67] and suggested a dissociation between the levels of sFas and sFasL. Further longitudinal studies are warranted in HBV-and HBV/HIV-infected patients to better understand the role sFas and sFasL are playing in HBV-mediated liver disease and whether there is any prognostic value in monitoring sFas and sFasL levels [46, 62, 68].

Soluble PD-L1 was similarly significantly (*P* < 0.005) higher in HBV/HIV coinfection compared to controls (Figure 1 and Table 2). In chronic viral infection, including HBV and HIV, persistent exposure to high concentrations of viral antigens leads to T-cell exhaustion. PD-1/PD-L1 interaction plays a critical role in T-cell exhaustion [69, 70]. In HBV infection, PD-1 expression on T cells correlates with viral load [38] and PD-L1 expressed on infected hepatocytes [71-74]. In addition to membrane-bound PD1 and PD-L1, there are circulating soluble PD-1 (sPD-1)[31] and soluble PD-L1 (sPD-L1) [75, 76]. Several lines of evidence implicate a role for the soluble forms in regulating the PD-1/PD-L1 pathways [77, 78].

In chronic viral infection, viral-specific T-cell reactivity is weak or absent (exhausted immune response) and characterized by poor cytotoxic activity, impaired Th1 cytokine production, and sustained expression of multiple inhibitory receptors, such as PD-1 [72, 79, 80]. Many studies have validated the existence of T-cell exhaustion in chronic viral diseases [79-81] and cancers [82, 83]. Blocking of the inhibitory receptors with their ligands leads to the development of more effective immune responses. For example, PD-1 blockade has already been proven to restore the functional activity of HCV-specific [84, 85] and HIV [86]-specific CD8^+^ T cells and improved the immunological control of tumors in humans [87]. PD-1 blockade can increase IFN-γ production in cells derived from HBV-monoinfected and HBV/HIV-coinfected patients as well [88].

There is accumulating evidence of a complicated interplay between the Fas/FasL pathway and PD-1/PD-L1 pathways. For instance, the neutralizing antibodies to PD-L1 and FasL significantly reduced the suppressive effect on T-cell proliferation [89]. However, only anti-PD-L1 antibody partially restored early T-cell activation. Anti-FasL antibody, but not anti-PD-L1 antibody, reduced apoptosis of activated T cells indicating that FasL molecule plays a role in inducing apoptosis of activated T cells. Therefore, the presence of different effects of PD-L1 and FasL molecules on T-cell activation and apoptosis of activated T cells suggests that these 2 molecules influence T-cell responses at different stages [89]. Only in the group with HBV monoinfection was a significant correlation observed between sFasL/PD1and sFas/PDL1. These data suggest that HBV-monoinfected and HBV/HIV-infected patients have different biomarker profiles that may relate to different immune responses and disease pathogenesis processes.

TGF-β1 was significantly higher in HBV monoinfection compared to healthy controls. TGF-β plays a major immunological role during HBV viral infection through both direct and indirect mechanisms. TGF-β directly stimulates hepatic stellate cells to synthesize and deliver extracellular matrix molecules such as collagens, fibronectin, and laminins within the stromal milieu [90]. There is also a strong cross talk among TGF-β and the tissue extracellular matrix components. TGF-β is stored in the matrix as part of a large latent complex bound to the latent TGF-β binding protein (LTBP), and matrix binding of latent TGF-β complexes is required for adequate TGF-β function. Once TGF-β is activated, it regulates extracellular matrix remodeling and promotes fibroblast to myofibroblast transition, which is essential in fibrotic processes. Therefore, within the liver, TGF-β is pro-fibrogenic. TGF-β also exerts immunosuppressive activity by inhibiting the host immune response. It is released by T cells, and it inhibits the secretion of TNF-α, IFN-γ, and other interleukins [91]. It also serves as a differentiation factor for T-regulatory cells [92], and its increase may be playing a significant role in the reduction of inflammation within the liver. The significant positive correlations between ALT and AST with TNFα and the significant negative correlations with TGF-β suggested a distinct role for each of those 2 cytokines in liver inflammation.

To evaluate the relationship and the potential interactions among the immunological biomarkers we examined the cluster correlation coefficient of the tested markers in the groups with HBV, HBV/HIV coinfection, and in healthy controls. The cluster coefficient profile analyses revealed distinct profiles for each group. In HBV monoinfection, IFN-γ-IL-2; IL12p70-IL-10-IL-6- TNF-α; and GM-CSF-IL1β are clustered together (Figure 2B). In contrast, the HBV/HIV coinfection group has a different unique profile with GM-CSF-IL-4- IL-2-IFN-γ-IL12p70; and IL-7-IL-10 -IL1-β grouping together (Figure 2C). The control group also has a unique profile with IL-10-sPD-1; IL-13- IL4-IL5-IL-1β-IL2-GM-CSF-IFNγ-IL12P70, and IL6-TNFα all grouping together (Figure 2A).

Cluster correlation of the apoptotic and exhaustion markers in the sera of the enrolled patients identified at least 2 independent apoptotic pathways. The first involved PD-1/PD-L1 and both FasL and TGFβ pathways. The second involved Fas and TNFα. The complex interaction between those 2 different pathways in HBV and HIV will be investigated in future studies.

The presence of unique biomarker profiles for each group is notable. The utilization of those unique biomarker profiles for disease progression is promising and will need further investigation. Additionally, the association of high levels of sPD-L1 and sFas with HBV infection suggests that targeting the PD-L1/PD-1 and/or Fas/FasL molecules in HBV-infected patients may be of therapeutic benefit in boosting the immune responses against HBV leading to viral clearance and/or functional cure.

One of the limitations of our study, is its cross-sectional design. Furthermore, we did not have PBMCs or follow-up samples from the enrolled patients. Additionally, the number of studied individuals is relatively small, and the control group was not matched to HBV and HBV/HIV groups for age and race. However, this descriptive study will be followed with a longitudinal study to test unresolved hypotheses.

In summary, HBV-infected patients had a unique biomarker clustering profile comprising IFN-γ, IL12p70, IL-10, IL-6, and TNF-α that was distinct from the profile of the healthy controls and from those with HBV/HIV coinfection. Longitudinal evaluation that better characterizes these markers in patients during different immunologic stages of the HBV natural history appear warranted.
